# Extrinsic goals benefit capitalism but not well-being. Rethinking the economy’s goal for a healthier future

**DOI:** 10.1093/heapro/daae090

**Published:** 2024-09-25

**Authors:** Robert J Noonan

**Affiliations:** Faculty of Health and Wellbeing, University of Bolton, Deane Road, Bolton BL3 5AB, UK

**Keywords:** capitalism, well-being, inequality, economic growth, health

## Abstract

The dramatic rise in non-communicable diseases around the world but notably in high-income countries like the UK is a manifestation of a global economic system—capitalism—that prioritizes wealth over health. A decade ago, the former WHO Director-General, Margaret Chan highlighted how ‘efforts to prevent non-communicable diseases go against the business interests of powerful economic operators’ [United Nations. (2013) Take Action for the Sustainable Development Goals. https://www.un.org/sustainabledevelopment/sustainable-development-goals/ (last accessed 16 February 2024)]. While there is a growing literature on how politics and economics influence population health—for better or worse—less attention has been given to exploring how economic systems like capitalism influence people’s psychological well-being. To fill this gap, the following article examines how the continued pursuit of economic growth under capitalism (neoliberal free-market forms especially) impacts well-being through challenging basic psychological needs for security, autonomy, competence and relatedness. In doing so, I hope to shed important light on the sources and possible solutions to our growing health and social problems, and stimulate a conversation on how to achieve a healthier future for us all.

**Contribution to Health Promotion**

- Growing the economy has become a higher priority than protecting and improving the health of all.
- Capitalism in its current form challenges basic psychological needs for security, autonomy, competence and relatedness which are essential nutrients for well-being.
- The relentless pursuit of economic growth under capitalism is at odds with the United Nations’ Sustainable Development Goals to achieve a healthier, more socially just and more sustainable future.
- We need an economy that prioritizes health over wealth so that everyone receives a fair slice of the cake and has the opportunity for a healthy and meaningful life.

## INTRODUCTION

Psychological well-being (referred to as ‘well-being’ herein) can be examined on many different levels, from the individual level right through to the societal level. At the individual level, well-being stretches beyond hedonism (i.e. happiness) and the subjective experienced state of ‘feeling well’—which comprises life satisfaction, positive effect and negative effect (Diener, 1984). It also encompasses eudemonia, which centres on ‘doing well’ (i.e. flourishing; eudemonic well-being)—including whether a person feels in control and makes choices that influence their well-being. Ryan and Deci (2001) defined this as a ‘fully functioning’ person. There are many theories of eudemonic well-being. Each of which offers its own distinct vision of what contributes to a *life well lived* and a *fully functional state*. While the use of terminology is varied, what is strikingly clear is the shared commonalties between them all (Martela and Sheldon, 2019).

**Fig. 1:**
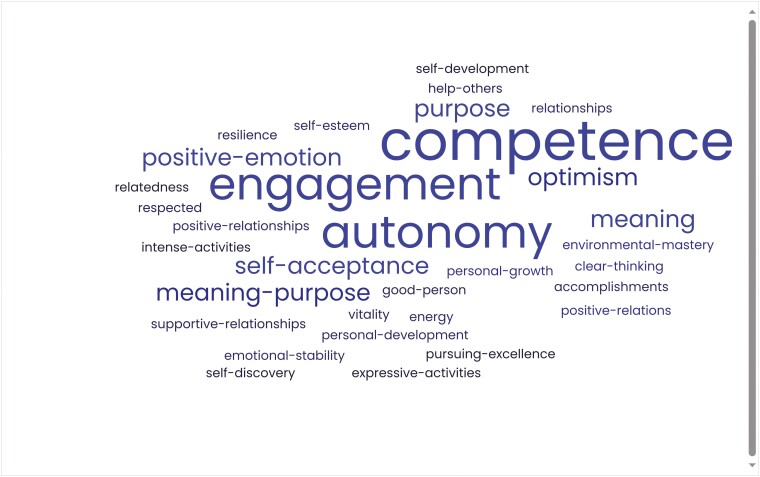
Visual representation of common themes in eudemonic well-being theories.

To illustrate this point, the individual elements from each of the eight most influential eudaimonic well-being theories (see Ryff, 1989; Deci and Ryan, 2000; Tennant *et al*., 2007; New Economics Foundation, 2008a; Diener *et al.*, 2010; Waterman *et al*., 2010; Seligman, 2011; Huppert and So, 2013), identified by Martela and Sheldon (Martela and Sheldon, 2019), were pooled together, and a word cloud was created to visually represent the most common elements (Figure 1). The purpose of this exercise, and indeed the article more broadly, is not to review or critique the theories in any specific way. But rather to highlight the synergies between them and illustrate how neoliberal free-market forms of capitalism (referred to as ‘capitalism’ herein; which views competition as the defining characteristic of human relations and promotes competition through deregulating capital markets, removing trade barriers, cutting taxes, eliminating price controls and reducing state influence in the economy through privatizing public services and austerity policies)—that relentlessly pursue economic growth, practiced in high-income countries like the UK and USA—challenges citizens’ psychological need for security as well as the pursuit of eudaimonic motives and activities (i.e. values, goals, practices)—the very things that promote and support subjective well-being (Martela and Sheldon, 2019).

Building on Deci and Ryan’s self-determination theory (Deci and Ryan, 2000), Kasser (Kasser, 2009) and Sheldon *et al*. (Sheldon *et al*., 2001) revealed that there are at least four psychological needs key to a person’s satisfaction with life and well-being. Humans need to first feel *safe and secure* and have confidence that they are able to meet their basic survival needs. Secondly, humans need to feel that they are autonomous—that is, they are independent, can regulate their own behaviour and can resist social pressures. Thirdly, humans need to feel competent—that they possess a sense of mastery and positive self-regard, and trust in their ability to engage in all that they care about. The fourth need, for relatedness, supportive relationships, positive relations—call it what you like—humans are social animals; they possess a hardwired innate drive and desire for warm, satisfying contact with others and suffer when they are socially excluded and lonely (Ryff, 1989; Kasser, 2009).

Our health including our mental health is greatly influenced by our social and built environment and our lifestyle behaviours (Braveman *et al.*, 2011; Scambler, 2012; Compton and Shim, 2015; Patel *et al*., 2018a). The same holds true for satisfying the four psychological needs highlighted above (Deci and Ryan, 2000, 2008; Kasser, 2009). In taking a needs-based approach, the remainder of the article will examine the extent to which our economic system—capitalism—is conducive to satisfying these four psychological needs.

In the first section, emphasis will be placed on showing how the values of capitalism—profit, competition, individualism and self-interest—foster in people an extrinsic orientation and external reinforcement of goal pursuits (i.e. wealth, power and status). In doing so, they devalue and ‘crowd out’ intrinsic goals (e.g. pursue own interests and personal growth) and non-economic activities which according to self-determination theory facilitate the experience of autonomy, competence and relatedness and foster well-being (e.g. Deci and Ryan, 2000, 2008). In the second section, the aim will be to first demonstrate how the unequal, unjust, exploitive and insecure nature of capitalism challenges the achievement of security for many, but especially the poorest in society. Secondly, it will show how the materialism promoted under capitalism only functions by preying on people’s emotions, continually stimulating in them anxiety and insecurity that will never truly be relievable by purchase.

## THE NEED FOR SECURITY, AUTONOMY, COMPETENCE AND RELATEDNESS

### Having rather than being and deficits over strengths

Capitalism is not just an economic system based on the private ownership of the means of production for profit (Smith, 1776). It is also a social system; it governs how our social relationships are both organized and experienced (Butler, 2019). In advanced capitalist countries with neoliberal free-market economies (e.g. UK and USA), strong support is given to individual private property rights, free functioning markets, free trade and the rule of law. Privatization, deregulation and competition are held to be primary virtues in order to boost productivity, reduce consumer costs and drive down the tax burden. Such an ideology promotes self-advancement and extrinsic values centred on hedonism (e.g. pleasure gratification for oneself), achievement (e.g. personal success) and power (e.g. social status, control over people and resources; Schwartz, 1992, 2012; Harvey, 2005).

Capitalism is effective at generating wealth and higher taxable incomes, which can be used to invest in public goods and promote equitable access to health without compromising human freedoms (Berdine *et al.*, 2018; de Soysa and Vadlamannati, 2021). However, the adoption of free, unregulated markets comes at the expense of inequality and concentrated wealth and power (Hathaway, 2020; Lansley, 2021; Hope and Limberg, 2022). The USA is the richest nation on earth, but one of the most economically unequal in the developed world. Not only does the USA record relatively poor life expectancy, deaths of despair (i.e. suicides and drug overdose) have risen dramatically in recent years (Alvaredo *et al*., 2018; Case and Deaton, 2020). The large variation in inequality and health measures across capitalist countries is thought to driven by differences in social policy (Wilkinson and Pickett, 2010; Ballas *et al*., 2014; Piketty, 2014; Tsugane, 2021).

Despite its downfalls, capitalism retains its grip on the public’s mind and the decisions of governments around the world (Rutar, 2024). Many countries have oriented towards free markets as measured by indexes of economic freedom, believing that it delivers more freedom, prosperity and life satisfaction. There are indeed merits to such claims (Graafand, 2020; Aydan *et al.*, 2022). After all, many people from around the world continue to migrate to high-income countries in search of improved security and living standards (European Parliament, 2024). However, what is often missing from the ‘freedom’ debate is the question: freedom for whom? Undeniably, one person’s freedom comes at the expense of another’s. It is possible for example for the freedoms of companies to greatly impinge upon those of workers and citizens in general (Stiglitz, 2024). Similarly, we must not confuse one of capitalism’s core values: individualism with autonomy and freedom. As Wright (Wright, 2019) asserts, while capitalism promotes the development of both freedom and democracy, it actually obstructs the fullest possible realization of such values because the decisions that shape society under capitalism are made by those who hold the wealth; those that do not have to work for them to survive—that is neither freedom nor democracy. Furthermore, the materialistic values that permeate society under capitalism reduce citizens to cloned consumers, who live under the illusion that they make their own choices, when in fact they are being influenced and persuaded to think and behave alike (i.e. conform; Cialdini, 2007; Verhaeghe, 2014).

In his landmark book, *Propaganda*, Edward Bernays argued that: ‘The conscious and intelligent manipulation of the organized habits and opinions of the masses is an important element in democratic society’. Chomsky (Chomsky, 2002) contends that as societies become freer and more democratic, governments lose the capacity to rule by force and instead turn to techniques of propaganda to influence public perception and opinion (see Lippmann, 2010). Materialism is a form of propaganda that fosters conformity, undermines critical thinking and has side effects for individuals and wider society (Kasser, 2003). Such consequences were detailed in *The Lonely Crowd*, published almost 70 years ago, in which the author, David Riesman, described how America was becoming an increasingly self-conscious society (Riesman *et al.*, 1950). He contended that the pressure to conform was not only challenging self-expression and individual autonomy, it was creating a widespread sense of alienation and loneliness among the population.

The widespread materialism propaganda under capitalism is forcing society to become more and more materialistic. As a consequence, living is becoming less about ‘being’ and more about ‘having’ (Fromm, 1976). Materialistic values and goals are great for capitalism but they damage psychological well-being (Dittmar *et al*., 2014; Dittmar and Isham, 2022). Tim Kasser has written extensively on this topic (see Kasser, 2003, 2009, 2011, 2016, 2017; Kasser and Brown, 2003; Kasser *et al*., 2007; Kasser and Sheldon, 2009). Across a series of experimental studies, Kasser *et al*. (Kasser *et al*., 2014) revealed that people’s well-being declines as they place relatively more importance on materialistic values and goals. Building on Grouzet *et al*.’s (Grouzet *et al*., 2005) and Schwartz’s (Schwartz, 1992) work on values, aspirations and goals, Kasser (Kasser, 2016) has gone on to show how when materialism takes a more central role in people’s lives—when they pursue extrinsic goals like status, wealth, image and possessions—intrinsic goals which are both satisfying and promote well-being (i.e. self-acceptance, personal growth and affiliation) become ‘crowded out’. Schwartz’s value model (Schwartz, 1992) illustrates how people who possess high materialistic values are more likely to place low importance on self-direction, benevolence and universalism values. These values encompass independent thought and self-respect, social justice and being supportive to others (Public Interest Research Centre, 2011; Schwartz, 2012).

A consequence of capitalism is that it devalues many non-economic activities which are proven to benefit health and well-being. This is because capitalism only values what it needs—money; the activities it does not need—the non-economic ones—hold little value. The five ways to well-being—connecting, being active, learning, taking notice and giving (New Economics Foundation, 2008b)—are non-economic activities that fit squarely with the intrinsic goals and values that Grouzet (Grouzet *et al.*, 2005), Schwartz (Schwartz, 1992) and Kasser (Kasser, 2003, 2009, 2011, 2016, 2017) have described. They are the priceless activities that are satisfying to pursue because they promote independent thought, goal-setting, self-respect (i.e. self-direction and self-acceptance), relationship building and empathy towards others (i.e. affiliation and benevolence) which enable people to experience a sense of purpose and meaning. Goals of building and maintaining social relations, feeling autonomous and competent, and setting goals for living and personal growth appear critical to positive psychological functioning (Ryff, 1989; Ryan and Deci, 2017). However, they compete against the interests of capitalism and corporations. Capitalism promotes and endorses what is productive and profitable rather than what is healthy because money is how the score is kept. In the leisure time that people do have available to them, it makes more sense [economically] for people to pursue activities and experiences that contribute the most to gross domestic product (GDP), even if this leads to people’s well-being being compromised. Society does what the economy values. Capitalism values status-seeking and career, financial and material success; therefore, people strive to attain these things (more than the five ways to well-being for example), because it defines their social standing (i.e. status) and influences how they are viewed and rewarded (Kasser *et al*., 2007).

Self-accepting people possess strong feelings of autonomy and competence. They possess a positive attitude towards themselves, resist social pressures and evaluate themselves by their own standards (Ryff, 1989; Ryan and Deci, 2017). But here is the thing—the economic system does not want people to feel content, satisfied and secure. Because there is more money up for grabs for companies when people feel inadequate and insecure. Companies use fear-based advertising to drive consumer behaviour. They are exceptionally good at persuading and using ‘dark nudges’ (Petticrew *et al*., 2020; Corcos, 2023; Kuyer and Gordijn, 2023). They use proven techniques like MINDSPACE (Dolan *et al*., 2012) to play on people’s fears and insecurities, which grips attention, promotes a higher standard of living and creates a sense of urgency to spend. In his book, *The Affluent Society*, which was published in the late 1950s, John Kenneth Galbraith (Galbraith, 1958) highlighted how advertising is not only hostile to, but subverts individual autonomy by making up people’s minds for them.

The materialistic values and goals which are heavily promoted in advertisements including image, popularity, wealth and material success relate negatively with self-esteem and self-worth (Grouzet *et al*., 2005; Kasser *et al*., 2007; Consiglio and van Osselaer, 2022). People scoring high on materialistic values compare themselves with others more often and are more likely to be concerned about how they are viewed in the eyes of others which can intensify feelings of inferiority (Kim *et al*., 2017). After all, comparison is the thief of joy (McCarthy and Morina, 2020; Goldberg, 2023).

Fear and negativity are other thieves of joy. The media like all companies are out to make a profit so they selectively report on stories that create the most drama and fear because this is what sells best. For instance, we hear a lot about wars, terrorism, natural disasters and homicides. But these terrible events kill only a fraction of people yearly (Roser, 2021). Information like this intensifies feelings of anxiety and results in citizens having an unbalanced world view. We hear less about the millions of people killed in transport accidents; the almost doubled number of people that take their own life than die from homicide; the many millions that die due to malnutrition and obesity (Dai *et al*., 2020; Larsson and Burgess, 2021; Ward *et al*., 2022), or from not being active enough (Katzmarzyk *et al*., 2022; World Health Organization, 2022). That is because information like this is not all that attention grabbing or hysteria inducing.

Another downfall of such distraction is that it obscures from the public the ‘real’ societal problems we face like meeting everybody’s basic needs, and information concerning the things that really make people happy and healthy. This includes factors like the social determinants of health (Health Foundation, 2024), appreciating our strengths and *being*, being curious and being mindful. Appreciating the good (i.e. gratitude; Höfer *et al*., 2020; Weziak-Bialowolska *et al.*, 2023) and celebrating our strengths are some of the proven ways to resist capitalism and enhance well-being (Wood *et al.*, 2010; Jans-Beken *et al*., 2020; Kirca *et al.*, 2023). Using and boosting our existing strengths and skills and learning new ones is beneficial to well-being because it raises self-esteem and feelings of competence (Ghielen *et al.*, 2018). When people are aware of their strengths and passions, it is easy to find purpose and meaning and achieve regular engagement. This state is what Csikszentmihalyi (Csikszentmihalyi, 1990) named flow; it refers the psychological condition of being cognitively and emotionally absorbed in an activity.

Being physically active is one way to experience flow and it satisfies other psychological needs. We have shown this in our own work (Hall and Noonan, 2023). However, while physical activities including walking are great for health and well-being (Hanson and Jones, 2015; Kelly *et al*., 2018; Ma *et al.*, 2023), they are no good for capitalism. Because they are (financially) free and do not raise the GDP all that much (see Noonan, 2023, 2024). All economic systems including capitalism drive the social norms that underpin success criteria and individual values which in a capitalist economy like ours is very much measured in relation to career, financial and material status. It is difficult to slow down and lead an active, healthier and more sustainable existence when a bulk of the messages in all spheres of life are extrinsically underpinned, and challenge intrinsic motivations and non-material goals.

### Economic and psychological insecurity

Capitalism is disruptive which makes living under the system extremely insecure and precarious. Capitalism has to be disruptive and destructive in order to create the new tastes and new desires required to boost higher rates of consumption, spur on production and achieve growth. But this has social costs. We see this cost in the technological unemployment and precarious working conditions which not only harm psychological security but work against many of the United Nations’ Sustainable Development Goals (SDGs), including decent work and well-being for all (Maslow, 1943; Benach *et al*., 2014; Parr, 2022).

The capitalist economic system is programmed to benefit the wealthy and extract from those that are not (Byrnes and Collins, 2017). In her book, *This Changes Everything*, Naomi Klein (Klein, 2014) explained how ‘extractivism is a nonreciprocal, dominance-based relationship with the earth, one purely of taking’. However, capitalism’s taking does not stop with nature. As well as extracting wealth from the land and water it *grabs* from poor communities (Rulli *et al*., 2013; Sassen, 2014; Dell’Angelo *et al*., 2018; Lochery, 2022), it extracts wealth from human data (Grimshaw, 2018; Sadowski, 2019) and human labour without fair compensation or regard for the collateral damage that ensues (Selwyn, 2019).

Capitalist accumulation is heavily dependent on profit. Marx (Marx, 1867) described how the extraction of surplus value is key to the process of exploitation under capitalism. Decades later, Robert Tressell, in his semi-autobiographical novel, *The Ragged Trousered Philanthropists*, depicted the plight of the working poor in Edwardian England and illustrated how the wealthy *bourgeoisie* dominated workers’ lives by means of ‘the great money trick’ (i.e. Marxist theory of surplus value: workers create more value than they receive in wages; Tressell, 1914). The rules of the (capitalist) game have remained the same since the days of Marx and Tressell and enabled the wealthy few to accumulate vast fortunes at the expense of ordinary working people. For example, in 2020, the world’s 2000 or so billionaires held as much wealth between them as the bottom 4.6 billion people–equating to roughly 60% of the global population (Oxfam, 2020). These figures should not come as any great surprise. Because, intuitively, in order for [a minority of] wealthy members of society to lead a life of extravagance and abundance, there has to be a sizable majority that are dirt poor and destitute. We have arrived at this point because the gross exploitation of the working classes has led to perverse patterns of maldistribution; principally owing to government policies making it easier for the rich to get richer and harder for the poor to achieve what they deserve—better pay and conditions (Sumner, 2016; Lansley, 2021; Sullivan and Hickel, 2023).

For instance, policy makers in countries like the UK have continuously chosen to cut taxes for the rich which widens income and wealth inequality (Oishi *et al.*, 2018; Hope and Limberg, 2022; Lierse, 2022). They have decimated trade union power giving companies greater freedom to suppress worker wages (Hagedorn *et al.*, 2016; Ahlquist, 2017; Daniels, 2023). They have underfunded public services—the very things the most vulnerable in society depend on (UNISON, 2019). Austere policies like this create living and working conditions that degrade health and kill (Marmot *et al*., 2020; Walsh *et al*., 2022a, 2022b). That the UK Government are not only aware of the social costs of capitalism, but do little to mitigate them, shows how the concept of ‘social murder’, which Engels (Engels, 1845/2009) highlighted almost two centuries ago, in *The Condition of the Working Class in England*, continues to plague Britain today (Medvedyuk *et al.*, 2021).

To survive and thrive, the human body and mind need nourishing—just like any plant does. If you pot a plant, then restrict its water supply, feed it lousy soil and deprive it of sunlight, you would be a fool to think that it was ever going to survive or thrive. Like plants, humans are heavily dependent on social medicine—good conditions of life (i.e. adequate shelter from the elements) and nutritious food. To meet these needs, personal income—the product of hourly wage and the number of hours worked—has to keep pace with living costs. But this does not happen for everyone (Das, 2023). It did not happen when Engels (Engels, 1845/2009) and Marx (Marx, 1849) were critiquing the capitalist system in the 1800s; and it does not happen now (Joseph Rowntree Foundation, 2023a; The Trussell Trust, 2023).

Millions of people go to work and remain poor (Joseph Rowntree Foundation, 2023b). When people are poorly paid (so that company profits can soar) and cannot afford to purchase good-quality food, their body becomes undernourished and they fall ill. For people living in poverty, each and every day is a battle to survive and remain afloat. The economic insecurity they experience is psychologically distressing and damaging to subjective well-being because it results in a disproportionate share of physical (e.g. substandard housing, noise, crowding) and psychosocial stressors (e.g. lack of control, stigma and status anxiety; Sapolsky, 2004, 2005; Marmot, 2006). What is more, when wages are kept low, workers are forced to work longer hours or take on a second job (Scott *et al.*, 2020; McBride and Smith, 2022). This results in them having to sacrifice time that would otherwise be used for resting and recuperating which is important for mental health (Sato *et al*., 2020). When this happens, workers are not only financially poor—they are time poor. They are forced to forgo leisure activities and are deprived of quality social interactions with their friends, children and wider family—the affiliative activities that support health and well-being (Umberson and Montez, 2010; Ryan and Deci, 2017; Shafer *et al*., 2018).

The first of the United Nations’ 17 SDGs is to end poverty (United Nations, 2023). The target is incredibly difficult to achieve under capitalism because capitalism promotes economic inequality which results in poverty (and eliminable human suffering). Economic inequality is no accident under capitalism. It is one of capitalism’s basic needs—it needs it to survive and flourish; in the same way, humans need nutritious food. Indeed, for a wealthy few to have so much it requires the rest of society to have very little. As we covered earlier, capitalism is not concerned with benevolence and universalism values—the welfare of others, society and the planet. It is concerned with self-interest and views greed positively. Capitalism’s proponents believe that greed is good because it leads to profit, which then drives investment in new technologies and products, which leads to those that can afford them having more choice and a higher standard of living. Therefore, not only does it want to bake a bigger cake but it also wants to retain and consume as many slices as possible.

While high-income countries (e.g. UK and USA) record higher life expectancy than low-income countries, and do not experience the same kind of infectious disease burden as them (because of their stage in economic development; GBD 2021 Causes of Death Collaborators, 2024), they do in fact experience a greater burden of mental health problems (e.g. anxiety and depression). Strong evidence shows that this burden is exacerbated by income inequality (Ruscio *et al*., 2017; Patel *et al*., 2018b; Wilkinson and Pickett, 2018; GBD 2019 Mental Disorders Collaborators, 2022; Henking *et al.*, 2023). For example, in a meta-analysis of research on the link between income inequality and mental illness, Ribeiro *et al*. (Ribeiro *et al*., 2017) revealed that greater inequality is related with higher rates of mental illness, especially depression and anxiety disorders. This well-established link was vividly illustrated in the documentary film, *The Divide*, inspired by the work of Wilkinson and Pickett (Wilkinson and Pickett, 2010).

There are a couple of plausible reasons for why economic division degrades mental health and well-being. The first concerns the psychological response of individuals to their perceived status in the social order. Human perceptions are relative (see contrast effect; Cialdini, 2007). They are based on our own experiences and the experiences of others. With regards to the latter, we are deeply sensitive to social status—our position relative to others (Layard, 2005). When income inequality is high, this can result in widespread discontentment because individuals see themselves as losing out, even when they are affluent in absolute and relative terms. Higher status anxiety is thought to be more prevalent in countries with wider income differentials because they experience greater status competition and individuals place a high value on what others think of them (Pickett, and Wilkinson, 2010; Wilkinson and Pickett, 2018). Secondly, as income inequality rises, status differentials between individuals do too; which can adversely affect social mixing and cohesion across groups, thereby reducing levels of interpersonal trust (Layte, 2012; Layte and Wheler, 2014).

It is noteworthy that the burgeoning of psychological and emotional disorders in high-income countries has happened against the backdrop of growing economies, rising absolute incomes, and the implementation of evidence-based mental health interventions (GBD 2019 Mental Disorders Collaborators, 2022). To be clear, the rise in prevalence of such disorders are not considered the result of material comforts and a higher general standard of living per se, but rather the individualism, competition, social stratification and ensuing (social evaluative) stress people experience from their sense of losing out and falling behind even when winning (Wilkinson and Pickett, 2018). A similar paradox has been evidenced by Levine (Levine, 2008) who found that productivity and individualism have double-edged consequences; people living in highly individualized countries that have a faster pace of life (e.g. USA) experience a higher standard of living but their fast-paced existence creates stress which has consequences for health.

The materialism promoted under capitalism only functions by continually stimulating in people anxiety and insecurity that will never truly be fulfilled by purchase. Not only are the poor in society socially excluded, those that are able to *conspicuously consume*—the more affluent—are not fulfilled by materialism either (see James, 1998, 2007, 2008). The capitalism narrative—that consumption of more things confers real satisfaction and guarantees a fulfilled life—is challenged by experimental evidence showing that materialistic messages have negative effects on individual and societal well-being (Moldes and Ku, 2020). What is more, human wants are difficult to satisfy because of the contrast effect; the tendency for people to compare what they have (or do not have) against what others have (i.e. social comparison) and what they themselves have become accustomed to having (i.e. adaptation). The pursuit of individual success (relative to others) endorsed by capitalism actively encourages between person rivalries which amount to intrinsically unproductive zero-sum games (e.g. ‘I want more money or more things than my neighbours’). The battle for relative success and status has limited net gain for individuals and society as a whole, because for every winner, there must be a loser; the pursuit of advantage for ourselves results in a disadvantage on others. These psychological principles go some way to explaining why continued economic growth and rising incomes have not boosted most people’s happiness and contentment, once basic needs have been fulfilled (Lane, 2000; Layard, 2005).

### Working harder and for longer

GDP—the measure used to track economic growth—is largely made up of consumer spending (~60%; House of Commons Library, 2023). For capitalism to survive and flourish, people have to keep spending more. For people to spend more, they have to work more. When people work more, they have less time to be *autonomous*—to relax, to cook, to do household chores, to walk for leisure and walk for transit. They become reliant on *outsourcing*—paying others for the goods and services they are unable to provide for themselves because of their time deficit. Currently, we have a situation now where millions, perhaps even billions of people, spend their hard-earned money on provision to compensate for the health costs and meaningful physical, social and leisure pursuits they sacrifice through overwork (Soper, 2011).

There is a widespread feeling of time poverty across society. Lots of people report a growing list of things to do and never enough time to accomplish them (Hamermesh, 2014, 2019). This is important to the debate because long hours of work and time poverty have an adverse impact on health and well-being including life satisfaction, positive affect and negative affect (Kasser and Brown, 2003; Kasser and Sheldon, 2009; Giurge *et al*., 2020). Moreover, when workers work more intensely or work long hours, they are more stressed, they burn out and their health suffers (Maslach and Leiter, 2016; Hunt and Pickard, 2022). Psychological distress including stress is the primary cause of work-related ill-health. In Great Britain alone, there were almost 1 million cases of work-related stress, depression or anxiety in 2022/23, resulting in an estimated 17 million working days lost (Health and Safety Executive, 2023). Physical health is also compromised as evidenced by a recent global study showing that overwork contributes to over 700 000 stroke and ischaemic heart disease related deaths every year (Pega *et al*., 2021).

Chronic stress does not just have a direct impact on human health. It has an indirect effect too. When people are chronically stressed—unhealthy lifestyles are more likely to kick in. Lifestyles comprised of convenience ultra-processed foods, minimal exercise and consumption of harmful substances like alcohol and tobacco. All of which are direct risk factors for cardiovascular disease (Steptoe and Kivimäki, 2013; Kivimaki and Kawachi, 2015; Yusuf *et al*., 2020; Bays *et al*., 2021). It is common sense really. This is why the UK and other European countries have legislation to prevent workers from being forced to work more than an average of 48 hours each week (i.e. working time directive). Although the diehard capitalists would love nothing more than to tear it up; because rest and recuperation are the antitheses of capitalism.

There is currently great polarization between the number of hours people are working. Some people are working too many hours, others are working none at all, and a growing share are underemployed—engaged in precarious, meaningless work (Irvine and Rose, 2022; Frank *et al*., 2023; Jaydarifard *et al*., 2023). The competition for work among the working classes and the relationship between overwork, underwork and unemployment were described by Marx (Marx, 1867) in his seminal text, *Capital*. Marx stated: ‘The overwork of the employed part of the working class swells the ranks of the reserve [the unemployed], whilst conversely the greater pressure that the latter by its competition exerts on the former, forces them to submit to overwork and subjugation under the dictates of capital’. For Marx, this zero-sum game is core to capital accumulation. It is notable that when workers work more to earn more money, not only do they condemn the other parts of society to less work which can arguably make them less happy, they may also assert a negative social influence on their colleagues by encouraging them to work more too (Layard, 2005). Overtime, this can create a culture of long working hours and presenteeism which not only harms well-being but reduces productivity (Collewet and Sauermann, 2017; Wong *et al.*, 2019).

That a proportion of society are overworking and consuming excessively (Wiedmann *et al*., 2020; Barros and Wilk, 2021; Watts, 2023) while other citizens do not have access to stable work and struggle to meet their basic needs is one reason why a shorter working week has been advocated (New Economics Foundation, 2010, 2018; Campbell, 2014). Proponents of such a policy have suggested that a shorter working week, including a more equitable distribution of working hours, may contribute towards tackling other societal challenges including widening income and health inequalities (Marmot *et al*., 2020; Lansley, 2021; Marmot, 2024), widespread stress and mental health-related sickness absence (Health and Safety Executive, 2023; Office for National Statistics, 2023), and unsustainable levels of consumption, waste and carbon emissions (Autonomy, 2019; Ahlström *et al*., 2020; Mathai *et al*., 2021). Although the evidence base in this area is not yet well established.

Cutting working hours may, however, tackle the time deficiency many workers face which can deprive them of the opportunity to pursue activities and experiences that support well-being. A recent systematic review study found that reductions in working time resulted in positive improvements in work–family relations and well-being outcomes (Hanbury *et al*., 2023). Similarly, another systematic review study found that reductions in working hours resulted in lower stress and improvements in health behaviour outcomes (e.g. physical activity and sleep; Voglino *et al*., 2022). Moreover, a recent 4-day working week trial in Spain led to employees reporting lower levels of stress, higher satisfaction and reduced levels of commuting which contributed to improving air quality (World Economic Forum, 2023). Therefore, the available evidence suggests that when people work less, they utilize the time to engage in well-being–enhancing activities. A policy in support of reduced working hours is perhaps even more timely, given the predicted job losses due to automation and artificial intelligence (Parr, 2022). However, despite UK evidence showing that working less boosts worker productivity and well-being (De Neve, 2022), the UK Government are not in favour of widespread adoption (Butler, 2023); arguably because policies like this that encourage people to work less fly directly in the face of capitalism.

## TWEAKING THE RULES AND CHANGING THE ECONOMY’S GOAL FOR A HEALTHIER FUTURE

Capitalism is to the mind what rust is to iron; it has a corrosive effect and degrades slowly. Capitalism is never satiated. It is always hungry for growth. To achieve growth it endorses working, spending and *having* , rather than *being*, including being connected, being active, being curious, and above all—being content with who we are and what we have. The relentless push for economic growth and material gain in high-income countries like the UK challenges psychological well-being because it does not make room for universal psychological needs. According to self-determination theory, we crave for positive social relationships, and a sense of personal growth, meaning, purpose and belonging because these are core to our sense of self and worth (Deci and Ryan, 2000, 2008).

The pursuit of economic growth under capitalism is causing many health harms across society. But it is the inconvenient truth that few academics challenge and one that is heavily obscured from public discourse and debate. Aside from widening health inequalities and intensifying the obesity and physical inactivity crises (World Health Organization, 2022; World Obesity Federation, 2023), it is resulting in increased levels of insecurity, loneliness and status anxiety which contribute to psychological distress and adversely impact well-being (HM Government, 2018; Becker *et al.*, 2021; Clair *et al*., 2021; Pybus *et al*., 2022; Noonan, 2023, 2024). The rise in psychological distress and decline in life satisfaction are against the backdrop of a growing economy and ever-rising levels of material comfort (Collishaw, 2015; Daly, 2022; Zhang *et al*., 2023). This is just one canary in the coal mine. The other canary is the public expense that is being spent on responding to these health harms (HM Treasury, 2022), the harms that capitalism has, in many respects, created.

The economic approaches taken by governments not only drive social norms and thus the behaviours citizens display to fit in, they directly impact citizen’s lives in other ways because they influence policies regarding taxes, welfare spending and labour market regulations which influence the nature of jobs and wages as well as working conditions. The welfare state which encompasses income support, sickness and unemployment benefit, housing subsidies, healthcare and pensions provides citizens security and contributes to a well-functioning society (Chang, 2023). It is an essential mechanism alongside other regulatory policies to mitigate against the insecurity and health effects capitalism can cause in pursuit of innovation and growth (economic dynamism).

Capitalism in its current form need not be our future. Significant regulation and redistribution of income and wealth can be put in place to counteract the collateral damage of capitalism and reduce the risks people experience in life in terms of employment, income and health. Governments including that of the UK have in the past (following World War 2) attempted to correct the harms of capitalist markets including individual vulnerability to risks, the limited provision of public goods and services and the negative externalities of economic activity (Wright, 2019). Unlike neoliberal free-market policies which increase economic inequality, poverty and insecurity and adversely impact health including mental health (Schrecker, 2016; Viens, 2019; Becker *et al.*, 2021), social democratic models of governance are capitalist but they retain a strong emphasis on welfare, progressive taxation and public investment. Such approaches maintain economic progress while also keeping inequality levels relatively low and living standards (for all) high. Although not perfect, the Nordic countries (Denmark, Finland, Iceland, Norway and Sweden) offer evidence that the social and health costs of (neoliberal free-market) capitalism can be mitigated somewhat through government policies and regulation given their high performance in equality, social security and working environment as well as alternative measures of social progress such as the Human Development Index (composite measure of three human development markers: health, knowledge and standard of living; Hvid and Falkum, 2019; Martela *et al*., 2020; Torp and Reiersen, 2020).

Regulation is a country’s most powerful mechanism to tame the impacts of capitalism and positively change culture and health behaviour at scale. Sanitary reform and banning smoking in public spaces are both cases in point. There is evidence to suggest that restrictions on food advertising to children are effective (Dillman Carpentier *et al*., 2023). Recently, the restriction of junk food advertising across the London, UK transport network led to reductions in cases of obesity, diabetes and cardiovascular disease (Thomas *et al*., 2022). A similar approach aimed at tackling corporate advertisements promoting materialistic values could be the next big public health advance.

Taming capitalism through tweaking the game’s rules (i.e. regulation and redistribution) would no doubt have societal benefits and is likely to be the more achievable strategy at least in the short term—but changing the economy’s goal has the potential for far greater long-term gains especially where health, well-being and sustainability are concerned. We need to move away from an economy that is unfair, insecure, unhealthy and extractive to one that is fair, healthy and sustainable. We need an economy that prioritizes health over wealth. Only then will it be realistically possible for governments to fully support their citizens to pursue the eudemonic goals and activities that support a fully functioning life—and in doing so achieve health and well-being for all. Human development frameworks like the capabilities approach based on the work of Amartya Sen (Sen, 1999) and Martha Nussbaum (Nussbaum, 2011) emphasize the promotion of social justice and have been advocated as guiding frameworks for the promotion of mental health and well-being and the achievement of the United Nations SDGs (White *et al.*, 2016; White, 2020; Seckler and Volkert, 2021). In contrast to traditional economic approaches which place high importance on economic indicators (i.e. income and consumption), the capability approach recognizes multiple dimensions of quality of life. The approach contends that an individual’s well-being is best evaluated based on their capability to pursue the goals and activities which they value (Sen, 2009). In such a context, the purpose of social progress and the goal of nations should be *human development*, to create environments that enable people to live healthy and meaningful lives.

Consistent with the United Nations’ SDGs, the capability approach adopts a multifaceted perspective on social progress and poverty in particular. However, embedding a capability approach into both regional and national developed is dependent on joined up thinking and policy objectives being valued and shared by all public sector stakeholders especially (i.e. planning, education, health etc.). Good practice examples are evident in the UK such as Wales’ *Wellbeing of Future Generations Act* which centres on seven connected well-being goals (see Jones, 2022). However, almost 20 years has passed since *The Health in All Policies* concept was first introduced, which encouraged intersectoral collaboration to improve health and health equity through action on the wider determinants of health. In the years since, the UK in particular has witnessed widening health inequalities, increased rates of mental health and chronic disease and burgeoning healthcare costs (Marmot *et al*., 2020; Marmot, 2024). Moreover, it has proven difficult engaging all sectors—with short-term economic benefits often remaining favoured at the expense of long-term health and societal costs. UK doctors recently highlighted how population health and well-being has deteriorated due to rising in-work poverty, weakened public services and a broken social safety net (British Medical Association, 2022). Perhaps now is the right time for a new bi-directional approach—an approach that places health and well-being at the forefront of all regional and national policies, so that all sectors of society benefit (Greer *et al*., 2022).

## Data Availability

No new data were generated or analysed in support of this research.
